# Supplementary material for: The interactome of intact mitochondria by cross-linking mass spectrometry provides evidence for coexisting respiratory supercomplexes
Source: Mol Cell Proteomics. 2017 Dec 8;17(2):216–32. doi: 10.1074/mcp.RA117.000470 (PMC5795388; doi:10.1074/mcp.RA117.000470)
Supplement: Supplemental Data [file supp_RA117.000470_133922_0_supp_23978_fzffwf.zip › spectra_annotation/mito_spectra_annotation/400-1-9-1-6-1.pdf]

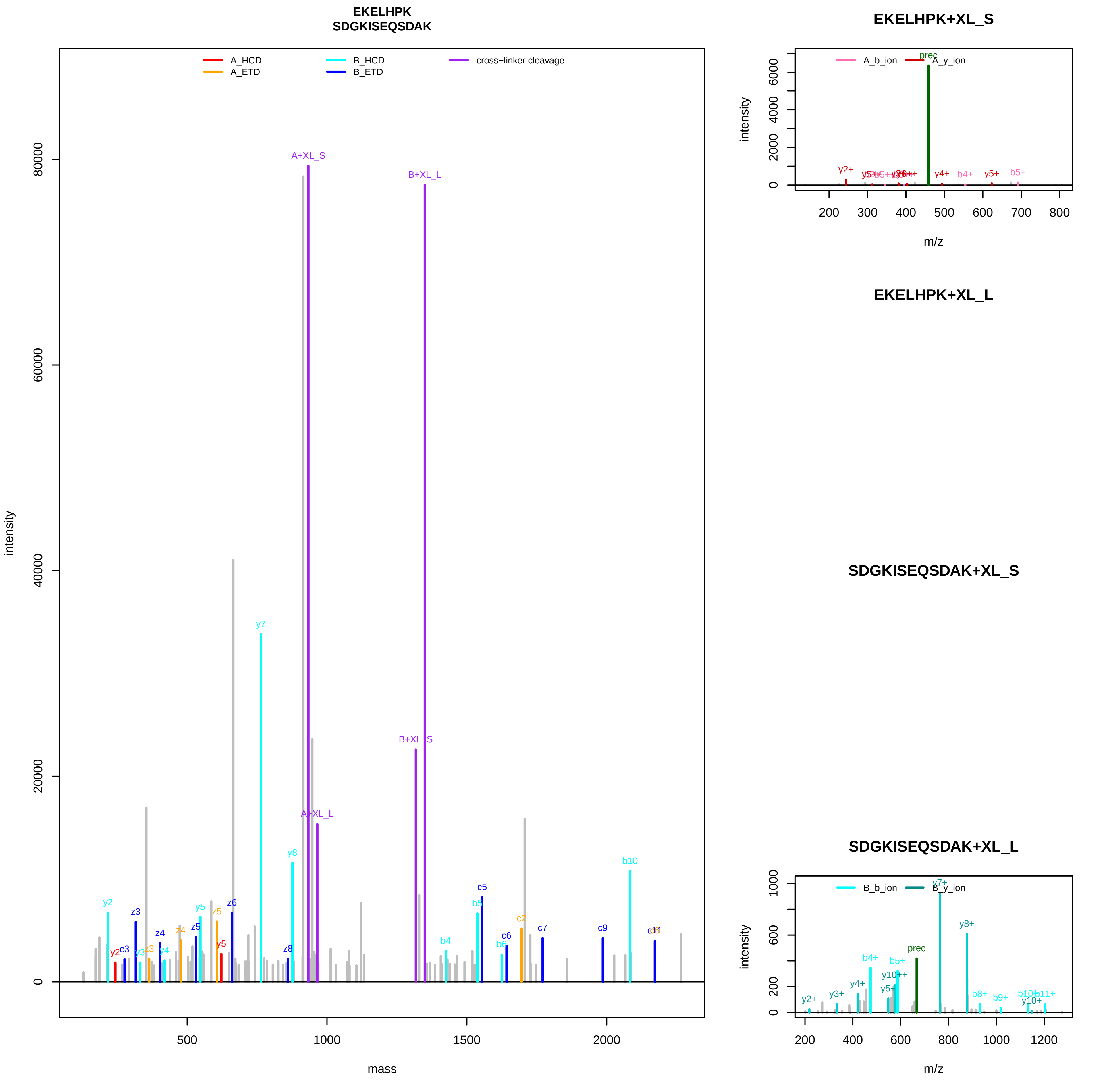
EKELHPK
SDGKISEQSDAK
A_HCD
A_ETD
B_HCD
B_ETD
cross−linker cleavage
A+XL_S
B+XL_L
B+XL_S
A+XL_L
y2
y3
y4
y5
y7
y8
y2
y5
c3
z3
z4
z5
z6
z8
c2
c5
c6
c7
c9
c11
b4
b5
b6
b10
mass
intensity
0
20000
40000
60000
80000
500
1000
1500
2000
EKELHPK+XL_S
A_b_ion
A_y_ion
prec
y2+
y4+
y5+
b4+
b5+
m/z
intensity
0
2000
4000
6000
200
300
400
500
600
700
800
EKELHPK+XL_L
SDGKISEQSDAK+XL_S
SDGKISEQSDAK+XL_L
B_b_ion
B_y_ion
prec
y7+
y8+
y2+
y3+
y4+
b4+
b5+
y5+
y10++
b8+
b9+
b10+
b11+
y10+
m/z
intensity
0
200
600
1000
200
400
600
800
1000
1200
